# Quality and Readability of Online Patient Education Materials Related to Branchial Cleft Cysts

**DOI:** 10.7759/cureus.24287

**Published:** 2022-04-19

**Authors:** Benjamin S Daines, Winslo Idicula

**Affiliations:** 1 Otolaryngology, Texas Tech University Health Sciences Center, Lubbock, USA

**Keywords:** quality of health information, readability, branchial cyst, branchial cleft cyst, patient education material

## Abstract

Introduction

Branchial cleft cysts are the second most common congenital neck mass and can cause significant anxiety for patients and families despite their benign nature. Education through online patient education materials (PEMs) is critical for informing patients and reducing stress. We aimed to determine the content, quality, and readability of online PEMs related to branchial cleft cysts.

Methods

The search engine Google was used to collect the first 100 website results for the query “branchial cleft cyst.” PEMs were included and assessed for content, quality via the DISCERN tool, and readability via Flesch Reading Ease Score (FRES), Flesch-Kincaid Reading Grade Level (FKGL), Gunning Frequency of Gobbledygook (GFOG), and Simple Measure of Gobbledygook (SMOG).

Results

Twenty-six websites containing PEMs related to branchial cleft cysts were assessed. Most websites were from universities or medical centers and did not contain any media. The mean DISCERN score was 49.3 (SD: 11.1, Median: 52.5), the mean FRES score was 51.9 (SD: 12.1, Median: 54.0), the mean FKGL score was 10.35 (SD: 2.52, Median: 9.95), the mean GFOG score was 13.32 (SD: 2.52, Median: 13.00), and the mean SMOG score was 10.25 (SD: 1.83, Median: 9.95). DISCERN was not significantly correlated with FRES, FKGL, GFOG, or SMOG.

Conclusion

Online PEMs related to branchial cleft cysts are consistently written above the National Institutes of Health (NIH) recommended sixth-grade reading level and are often of unsatisfactory overall quality. Writers of online PEMs for branchial cleft cysts should consider the readability and quality of their materials to improve patient education and reduce anxiety.

## Introduction

Branchial cleft cysts are neck masses that occur due to anomalies in the development or involution of the branchial arches leading to the persistence of branchial remnants [1,2]. These masses can present in a variety of locations across the lateral neck depending on whether the first, second, third, or fourth branchial arch is involved. They represent the second most common congenital head and neck mass and the most common congenital lateral neck mass [3]. Most patients with branchial cleft anomalies present with a cystic mass, however, fistulas and sinuses are other possible presentations depending on the degree of involution which has occurred [4]. Branchial cleft anomalies most often present in children with branchial cleft cysts typically presenting slightly later in life [4]. Masses may become inflamed and tender following an upper respiratory infection and potentially present as a fluctuant abscess [5]. Branchial cleft cysts are benign lesions that respond well to surgical excision [5]. Despite the benign nature of branchial cleft cysts, neck masses can cause significant stress and anxiety for both patients and families [6]. Patient education and reassurance are critical for preventing anxiety and informing patients about branchial cleft cysts.

The use of the Internet has become nearly ubiquitous in the United States, with 93% of Americans using the Internet in 2021 [7]. Previous studies have shown that 72% of Internet users have searched for health conditions and information online, meaning a vast majority of Americans have used the internet for health purposes [8]. Nearly half of consumers of online health information have been influenced by the Internet to make doctors' appointments, with many having diagnostic or treatment-related questions [9]. While the Internet provides a great deal of medical information, there is concern regarding the unregulated nature of such information [10]. Online medical information in the form of patient education material (PEM) is often incorrect, biased, subjective, or outdated, which can prevent patients and families from making informed decisions [10].

There exists further concern regarding the readability of PEMs and how accessible they are to a general audience. Most health materials are written at a 10th-grade reading level or above, yet most adults read at an eighth or ninth-grade level [11]. The National Institutes of Health (NIH) advises that written healthcare material, like PEMs, should be written at a sixth-grade level or below with pictures or illustrations to promote health literacy [12]. Low health literacy has been associated with more hospitalizations and worse overall health due to a poorer understanding of diseases and treatments [13]. Therefore, improving the readability and quality of PEMs may improve health literacy and subsequent health outcomes.

Previous studies have investigated the readability and quality of various otolaryngologic diseases or treatments, including head and neck cancer, tonsillectomy and sleep apnea, and parathyroid surgery [14-16]. To our knowledge, no such study has investigated branchial cleft cysts, one of the most common causes of congenital neck masses [3]. Here, we conduct an analysis of the content, quality, and readability of online PEMs regarding branchial cleft cysts.

## Materials and methods

Search methodology

The search engine Google.com was queried for the phrase “branchial cleft cyst” on February 11, 2022 from Amarillo, Texas. The top 100 website results were collected without refinement and recorded in Microsoft Excel 2016 (Microsoft Corporation, Redmond, WA).

Inclusion and exclusion criteria

Websites that were PEMs about branchial cleft cysts were included. Websites were excluded if they were duplicates if they were scientific articles published in journals or books if they required subscription services if they were not written in English, if they only contained video or audio information, if they were exclusively post-operative information, or if they were surgical guides.

Website categorization

Websites that met inclusion criteria were categorized using a methodology set by Ni Riordain and McCreary [14]. Categories included affiliation (commercial, non-profit, governmental, and university or medical center), specialization (website entirely or partially related to branchial cleft cysts), and content presentation (no media used, images, or video).

Quality determination

The DISCERN Instrument was used to determine the quality of online PEMs [17]. DISCERN is a 16-item test with each category scored from 1 to 5, allowing a minimum score of 16 and a maximum score of 80. An item scored 5 has completely fulfilled the criteria, whereas an item scored 1 has not satisfied the criteria at all. DISCERN has three distinct sections with items 1 through 8 assessing the reliability of the website, items 9 through 15 assessing treatment information, and item 16 assessing the overall quality of the website. Included websites were scored using the DISCERN instrument by the primary author BD.

Readability determination

Readability was assessed using Flesch-Kincaid Reading Grade Level (FKGL), Flesch Reading Ease Score (FRES), Gunning Frequency of Gobbledygook (GFOG), and Simple Measure of Gobbledygook (SMOG) [18,19]. Text from PEMs was copied and pasted into the online WebFX Readability Test to quantify these metrics [20]. FKGL, GFOG, and SMOG all provide estimates of the number of years of formal education required to understand a piece of written information. A score of 8.0 would correlate to an eighth-grade reading level required to understand the text. The National Institutes of Health (NIH) recommends that PEMs be written at a grade level of 6 or below. FRES is scored on a scale from 0 to 100 with higher scores indicating the information is easier to read and lower scores indicating the text is complicated. The target FRES score for written material is 65 or above.

Data analysis

Data was imported into IBM SPSS Statistics for Windows, version 28 (IBM Corporation, Armonk, NY) for analysis. Results were summarized using means, standard deviations, and percentages. Descriptive statistics were conducted using linear regression with a two-sided p-value set at P < 0.05 for statistical significance. Results were displayed using tables.

## Results

**Figure 1:**
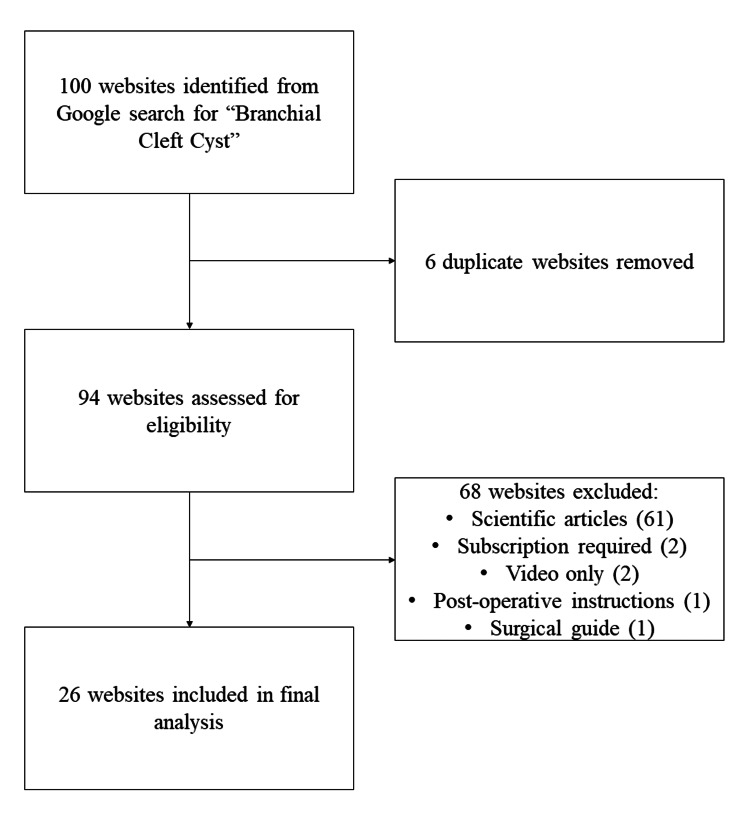
Flow chart demonstrating website inclusion and exclusion from the study

Of the 100 websites identified via Google search for “branchial cleft cyst”, 26 met the study inclusion criteria (Figure 1). Seventy-four websites were excluded because they were duplicate websites (6), they were formal scientific articles (61), they required subscription service to access (2), they were only videos without text (2), they were only post-operative instructions for the patient (2), or they were a surgical guide (1).

Each of the 26 websites that met the inclusion criteria for the study was differentiated based on affiliation, specialization, and content presentation (Table 1). The content type was excluded from analysis due to 25 of the 26 websites presenting medical facts, with only one presenting a human interest story. The most common website affiliation was with a university or medical center (13) followed by commercial (8) and non-profit (5) organizations. Most websites were entirely related to branchial cleft cysts (14) while fewer websites were partially related (12). Most websites did not use any media (19) but some used images (6) or video (1) alongside text.

**Table 1:** Categorization of articles according to affiliation, specialization, and content presentation

Category	Criteria	Number of websites (%)
Affiliation	Commercial	8 (30.8%)
	Non-profit organization	5 (19.2%)
	Governmental	0 (0%)
	University or medical center	13 (50.0%)
Specialization	Site entirely related to branchial cleft cysts	14 (53.8%)
	Site partially related to branchial cleft cysts	12 (46.2%)
Content presentation	No media used	19 (73.1%)
	Image	6 (23.1%)
	Video	1 (3.8%)

Website quality was rated using the DISCERN tool, which returned a mean score of 49.3 (SD: 11.1, Median: 52.5) out of 80. The range in DISCERN scores was 31 to 70. There are no cutoff scores used for DISCERN to indicate quality, but higher scores are associated with better information quality.

For assessment of website readability, FKGL analysis returned a mean of 10.35 (SD: 2.52, Median: 9.95) correlating to an average of a 10th-grade reading level. The FKGL score ranged from 5.3 to 15.4 indicating a grade-level range between fifth and 15th grade. Only 1 of the 26 websites (3.8%) read at a sixth-grade reading level or below. The mean score for FRES was 51.9 (SD: 12.1, Median: 54.0) which is defined as “fairly difficult to read” and correlates to a 10th to 12th-grade reading level. The FRES score ranged between 25.0 and 79.0. Only two of the 26 websites (7.7%) scored above the optimal score of 65. The average GFOG score was 13.32 (SD: 2.52, Median: 13.00) correlating with a grade level of 13 with a range between 8.3 and 17.5. The average SMOG score was 10.25 (SD: 1.83, Median: 9.95) correlating with a grade level of 10 with a range between 6.2 and 13.7. DISCERN was weakly positively correlated with FRES (r=0.012, p=0.955) and GFOG (r=0.004, p=0.986) while being weakly negatively correlated with FKGL (r=-0.052, p=0.802) and SMOG (r=-0.094, p=0.647).

## Discussion

Branchial cleft cysts are common congenital neck masses that can cause significant anxiety for patients and families despite their benign nature [6]. Online PEMs have the potential to reduce this anxiety and inform patients of branchial cleft cysts and their treatment. Furthermore, informed patients demonstrate better treatment compliance and health outcomes [21]. The quality of online PEMs related to branchial cleft cysts was measured using the DISCERN tool [17]. DISCERN rates health materials based on reliability, treatment information, and overall quality. DISCERN scores did not significantly correlate with any of the four measures of readability, indicating that website quality and readability were unrelated. There was significant variability in the quality of PEMs, evidenced by a wide range of DISCERN scores between 31 and 70. Previous internet quality studies on various otolaryngologic topics have demonstrated similar wide ranges in PEM quality [15,22]. This variety in PEM quality may bias or inadequately inform patients who only access a few websites regarding branchial cleft cysts. The lowest scoring DISCERN category in this study was “Is it clear what sources of information were used to compile the publication?” with a minority of PEMs citing sources. This may be attributed to half of the PEMs being written by universities or medical centers where the authors are often experts in the field. The third-lowest scoring category was “Does it describe the risks of each treatment?” with many PEMs neglecting to mention any. Branchial cleft cysts are often treated with surgical excision, so informing patients and families of potential risks is critical and should be stressed in future PEMs.

The readability of online PEMs related to branchial cleft cysts was assessed using FRES, FKGL, GFOG, and SMOG. Multiple assessments were used to account for different aspects of readability including sentence length, syllables per word, and a number of complex words [18,19]. All four assessments of readability placed the mean PEM at a grade level between 10th and 13th grade, significantly higher than the sixth-grade reading level recommended by the NIH [12]. FRES measured two websites reading at or below a sixth-grade reading level while FKGL measured one. Neither GFOG nor SMOG measured any websites at or below a sixth-grade reading level. With most adults reading at an 8th to ninth-grade level, these results indicate most people would be unable to comprehend the information presented in the majority of online branchial cleft cyst PEMs [11]. Previous studies investigating the readability of online PEMs for other otolaryngologic topics, such as nasal septoplasty and cochlear implants, have found similarly advanced reading levels [23,24]. To improve the readability of such PEMs, suggestions have been made to reduce the number of complex words, decrease sentence length, and format with bullet points [25].

Patient education may be negatively impacted by the lack of illustrations, pictures, videos, or other accompanying media in branchial cleft cyst PEMs. Previous studies have found pictorial health information to improve patient knowledge, especially in those with lower health literacy [26]. The NIH advises that PEMs include pictures or illustrations when appropriate [12]. Only seven of the 26 websites included any type of media beyond the article text. Branchial cleft cysts are a unique otolaryngologic complaint that often presents with a visually striking lateral neck mass. Future PEMs should aim to include pictures for patients to learn from and compare to so they can make more educated healthcare decisions.

This study was limited by the exclusive use of one search engine. While Google is the most popular search engine worldwide, utilizing additional search engines may have revealed additional PEMs that were not accessed for this study [27]. Furthermore, patients may use other search terms like “neck mass” when referring to a branchial cleft cyst which may lead to other PEMs. Future studies should aim to determine the quality and readability of other common neck masses. Clinicians creating online branchial cleft cyst PEMs should consider quality and readability to create materials that allow patients and families to make informed decisions.

## Conclusions

This study assessed the quality and readability of online branchial cleft cyst PEMs. On average, online branchial cleft cyst PEMs are written at a 10th to 13th-grade reading level, which is significantly higher than the sixth-grade reading level recommended by the NIH. Many branchial cleft cyst PEMs are of unsatisfactory overall quality. Clinicians should make a conscious effort to construct high-quality, readable PEMs related to branchial cleft cysts to reduce patient anxiety and provide them with the necessary knowledge to make informed medical decisions.
